# Development and Preliminary Field Evaluation of an Indirect ELISA for Detecting Tomato Yellow Leaf Curl Virus

**DOI:** 10.3390/v18070786

**Published:** 2026-07-19

**Authors:** Zeling Zhang, Yifan Liu, Xiangyu Zhang, Xianle Xue, Ting Xu

**Affiliations:** Beijing Key Laboratory of Biodiversity and Organic Farming, College of Resources and Environmental Sciences, China Agricultural University, Beijing 100193, China

**Keywords:** tomato yellow leaf curl virus (TYLCV), polyclonal antibody (pAb), plant virus detection, enzyme-linked immunosorbent assay

## Abstract

Tomato yellow leaf curl virus (TYLCV) is a major threat to tomato production, creating a need for sensitive, low-cost detection methods that can be applied to early symptomatic or low-viral-load samples. Recombinant antigen configuration may influence serological assay development, although the specific contribution of multiple-cloning-site (MCS)-derived intermediate sequences remains uncertain. In this study, a recombinant Trx-His-coat protein (CP) fusion antigen was produced using an MCS-free direct-fusion construct that retained the Trx-His tag while removing the MCS-derived intermediate sequence, followed by gradient refolding. No direct comparison with linker-containing, tag-cleaved, or tag-free antigen constructs was performed. The purified antigen was used to immunize rabbits and generate a high-titre polyclonal antibody (pAb). The resulting indirect enzyme-linked immunosorbent assay (ELISA) achieved a theoretical limit of detection of 1.8 ng/mL and an estimated pre-dilution equivalent the limit of detection (LOD) of 72 ng/mL after sample dilution. The assay showed favourable tolerance to crude tomato leaf matrices, with spike-recovery rates of 95.45–100.40%. In a preliminary evaluation using a balanced panel of 32 field-collected samples, ELISA absorbance correlated with droplet digital PCR quantification (*R*^2^ = 0.9819) and plant disease index values (*R*^2^ = 0.9774). Liquid chromatography–tandem mass spectrometry (LC-MS/MS) peptide mapping and AlphaFold2-based modelling were used only to provide preliminary computational context for antigen interpretation. The assay showed cross-recognition toward Tobacco curly shoot virus (TbCSV), indicating that it should not be considered strictly TYLCV species-specific. Therefore, this assay may support preliminary serological screening under the tested conditions, whereas molecular confirmation remains necessary when species-level identification is required.

## 1. Introduction

Tomato (*Solanum lycopersicum* L.) is widely cultivated worldwide because of its nutritional value and economic importance [1,2]. Greenhouse cultivation has expanded steadily to meet market demand and improve production efficiency [3,4]. However, high-density greenhouse production systems can also favour the spread of tomato diseases.

Tomato yellow leaf curl virus (TYLCV) outbreaks have been reported in major production regions [5,6]. TYLCV is a single-stranded circular DNA virus in the genus *Begomovirus*, family *Geminiviridae*, and is primarily transmitted by *Bemisia tabaci* [7,8]. Infected tomato plants commonly show leaf curling, chlorosis and severe stunting, which can cause substantial yield losses and economic damage [9,10]. Reliable, high-throughput diagnostic tools suitable for early field screening are therefore important for epidemiological monitoring and disease control.

Molecular diagnostic techniques, including quantitative PCR (qPCR) and droplet digital PCR (ddPCR), are widely used because of their high sensitivity [11,12]. However, these methods require nucleic acid purification, thermal instrumentation and trained personnel, which limits their use in local agricultural extension settings. By contrast, enzyme-linked immunosorbent assay (ELISA)-based detection remains useful for large-scale epidemiological screening because it is high-throughput, cost-effective and compatible with simple sample preparation [13,14]. A practical TYLCV CP-based indirect ELISA should provide adequate analytical sensitivity, reduced matrix interference, low cost, and operational simplicity. Compared with nucleic-acid-based assays, such an ELISA workflow can reduce dependence on DNA extraction and thermal instrumentation while remaining suitable for high-throughput preliminary screening.

Recombinant antigen configuration is one factor that may influence ELISA development. In heterologous expression systems, the coat protein (CP) coding sequence is commonly expressed together with fusion tags and vector-derived intermediate residues [15]. The effects of these intermediate sequences on folding, antigen presentation, and immunoreactivity may vary among individual constructs and were not directly evaluated in the present study.

Previous strategies have modified recombinant antigens through truncated expression or tag cleavage, although such modifications may also alter antigenic regions [16,17]. In this study, a multiple-cloning-site (MCS)-free direct Trx-His-CP fusion construct was used for recombinant antigen production. The cloning strategy is illustrated schematically in Figure S1. The Trx-His tag was retained, whereas the MCS-derived intermediate sequence between the tag and CP was removed. Here, the term “MCS-free” refers only to the construct architecture. Because no MCS-retaining or linker-containing construct, tag-cleaved antigen, or tag-free CP control was included, the present study does not establish an experimentally demonstrated advantage or superiority over alternative antigen configurations. Using the resulting polyclonal antibody (pAb), we established an indirect ELISA and evaluated its analytical performance, matrix tolerance and preliminary applicability in field-collected tomato samples. We also integrated liquid chromatography–tandem mass spectrometry (LC-MS/MS) peptide mapping with AlphaFold2-based structural modelling to provide a preliminary reference for putative surface accessibility and cross-recognition toward a related *begomovirus*. Overall, this study presents a preliminary recombinant antigen-based ELISA screening approach for TYLCV detection, with broader field validation still required.

## 2. Materials and Methods

### 2.1. Construction and Validation of Recombinant Expression Vector pET32a-CP

An MCS-free direct-fusion construct was generated by homologous recombination while retaining the N-terminal Trx-His tag. Using the TYLCV CP coding sequence (Accession No. ABG73017.1) as the template, the target fragment was amplified with Phanta Max Master Mix high-fidelity DNA polymerase. Homologous sequences of 15–20 bp were added to the 5′ ends of the primers (Supplementary Table S1) to match the terminal regions of the vector linearized with *BamH*I and *Xho*I. The purified PCR product was cloned into the linearized vector using the ClonExpress II One Step Cloning Kit (Vazyme Biotech Co., Ltd., Nanjing, China) at 50 °C for 15 min. The ligation product was transformed into *E. coli* DH5α competent cells, and positive clones were verified by colony PCR and Sanger sequencing to confirm the correct translational reading frame and the absence of mutations. A schematic representation of the MCS-free direct-fusion cloning strategy is provided in Figure S1.

### 2.2. Recombinant Protein Expression

The recombinant strain *E. coli* BL21(DE3)/pET32a-CP was cultivated in LB medium containing 100 μg/mL ampicillin at 37 °C until the OD_600_ reached 0.6–0.8. Protein expression was then induced with 1 mM IPTG. To optimize the recombinant protein yield, a one-factor-at-a-time (OFAT) approach was used to assess induction temperatures (16, 20, and 25 °C) and induction durations (12, 16, and 20 h). Bacterial cells were harvested by centrifugation and resuspended in lysis buffer containing 1 mg/mL lysozyme and 1 mM PMSF. Cells were disrupted by ultrasonication at 300 W for a total processing time of 20 min, with cycles of 30 s on and 30 s off under ice cooling to limit heat accumulation. The lysate was centrifuged at 12,000× *g* for 20 min to separate the soluble supernatant from the insoluble inclusion body fraction.

### 2.3. Target Protein Purification, Gradient Refolding, and Characterization

To obtain a soluble refolded preparation of recombinant Trx-His-CP fusion protein, the protein concentration was adjusted to 0.3 mg/mL, and the solution was transferred into 10 kDa molecular-weight cut-off dialysis tubing. Gradient dialysis was performed against PBS (pH 7.4) with stepwise decreases in urea concentrations (4, 2, 1, 0.5, and 0 M) at 4 °C for 12 h per step, allowing gradual removal of urea and imidazole. The dialysed protein solution was centrifuged to remove residual insoluble aggregates. The purity of the refolded recombinant antigen was then evaluated by SDS-PAGE and densitometric analysis using ImageJ, version 1.54g (National Institutes of Health, Bethesda, MD, USA).

The identity of the target protein was first verified by Western blotting with an anti-His-tag monoclonal antibody, followed by in-gel trypsin digestion of the excised target band and LC-MS/MS analysis. Mass spectrometry was performed on a Q Exactive hybrid quadrupole-Orbitrap mass spectrometer (Thermo Fisher Scientific, Waltham, MA, USA) coupled to an Easy-nLC 1200 system operated in data-dependent acquisition mode (Top 20). Raw data were processed using MaxQuant software (version 1.6.2.10), with carbamidomethylation of cysteine set as a fixed modification. Spectra were searched against a UniProt TYLCV database combined with the *E. coli* K-12 host proteome database. High-confidence peptide coverage was used to support the identity of the recombinant antigen. For undetected peptide segments, theoretical isoelectric point and grand average of hydropathicity were evaluated using ExPASy ProtParam (SIB Swiss Institute of Bioinformatics, Lausanne, Switzerland; accessed on 18 July 2026) to examine whether sequence properties might affect tryptic digestion or ionization efficiency.

### 2.4. Polyclonal Antibody Preparation and Affinity Purification

Two Japanese white rabbits (E25771 and E25772, aged 2–3 months, weighing 2.0–3.0 kg) were immunized with the purified recombinant Trx-His-CP fusion antigen. For primary immunization, 0.3 mg of fusion antigen was emulsified with complete Freund’s adjuvant (CFA) and injected subcutaneously at multiple sites on the back (the detailed immunization scheme is outlined in Figure S2). Three booster immunizations were then administered at 14-day intervals, each using 0.15 mg of protein emulsified with incomplete Freund’s adjuvant (IFA). Blood was collected 12 days after the final immunization, and antiserum was evaluated by indirect ELISA titration. The antiserum with the highest titre and most stable signal was loaded onto an antigen-affinity chromatography column. The antigen-affinity-purified pAb was eluted with 0.1 M glycine-HCl (pH 2.5), immediately neutralized with 1 M Tris-HCl (pH 9.0), and dialysed against PBS at 4 °C for 24 h. The purified pAb concentration was determined using a BCA Protein Assay Kit (Cat. No. P0012; Beyotime Biotechnology, Shanghai, China). Western blotting was performed using 1–50 ng recombinant Trx-His-CP fusion antigen at a working antibody concentration of 1 μg/mL to evaluate pAb specificity and detection limit.

### 2.5. Establishment and Evaluation of the ELISA System

An indirect ELISA was developed to evaluate the binding performance of the purified pAb. Checkerboard titration was first performed using recombinant Trx-His-CP fusion antigen as the coating antigen to optimize the CP coating concentration and pAb working dilution according to the signal-to-noise ratio (S/N). Under the optimized conditions, a pAb titration curve was generated using serial dilutions to characterize antibody titre.

A standard curve was then established using serial dilutions of recombinant Trx-His-CP fusion antigen. A four-parameter logistic (4PL) regression model was used to fit the relationship between antigen concentration and OD_450_. Based on 20 blank substrate wells, the blank-response threshold was calculated as the mean blank OD_450_ plus three standard deviations. The corresponding antigen concentration was then obtained by inverse prediction from the fitted 4PL calibration curve and was reported as the analytical limit of detection (LOD). To evaluate potential background reactivity toward the Trx fusion tag, recombinant Trx control protein was produced using the same bacterial expression system and purification workflow as the recombinant TYLCV CP antigen. This control protein was used as a negative control antigen in the specificity assay. Trx-directed background reactivity was assessed by comparing pAb response curves against the Trx control protein and the target Trx-His-CP fusion antigen.

### 2.6. Performance Evaluation of the Detection System

To assess matrix interference under standardized analytical conditions, crude homogenates of healthy tomato leaves were serially diluted (20.0%, 10.0%, 5.0%, and 2.5%) to construct a matrix background curve and determine the optimal sample dilution factor. Matrix effects were calculated using standard equations. Spike-recovery tests were then performed by adding recombinant Trx-His-CP fusion antigen to diluted matrices at 25.00, 100.00 and 400.00 ng/mL, and recovery rates were calculated to evaluate analytical accuracy. Assay precision was assessed using repeatability experiments. Intra-assay coefficients of variation (CVs) were calculated from three replicates within a single plate, and inter-assay CVs were determined across three independent experiments conducted on separate days.

For cross-reactivity analysis, Tobacco curly shoot virus (TbCSV) was included as a closely related *begomovirus* to evaluate cross-recognition within the same viral genus, whereas Tomato mosaic virus (ToMV), Tomato chlorosis virus (ToCV), and Cucumber mosaic virus (CMV) were selected as common tomato-infecting heterologous RNA viruses representing taxonomically unrelated viral groups. Accordingly, leaf samples infected with TYLCV, TbCSV, ToMV, ToCV, or CMV were analyzed, with healthy tomato leaves used as negative controls. Tissue homogenates were diluted to the optimized dilution factor before ELISA analysis. The positive cut-off threshold was defined as the mean OD_450_ value of healthy leaf extracts plus three times the standard deviations (*Cut*-*off* = *Mean_healthy_* + 3 × *SD_healthy_*). An individual sample was classified as ELISA-positive when its OD_450_ value exceeded the cut-off threshold. For graphical comparison, OD_450_ values were compared with the healthy leaf control using one-way analysis of variance (ANOVA) followed by Dunnett’s multiple-comparison test in GraphPad Prism, version 11.0.2 (GraphPad Software, LLC, Boston, MA, USA).

### 2.7. Field Sample Panel Construction and Disease Index Grading

For preliminary methodological evaluation, a total of 32 field-collected tomato leaf samples were assembled as a balanced sample panel. Samples were collected from tomato-growing sites in Beijing, China, during the winter of 2025 and the spring of 2026. The panel included four symptom-severity categories: healthy, mild, moderate and severe (*n* = 8 per category). Disease index (DI) scores were assigned according to visual symptom severity before ELISA and droplet digital PCR (ddPCR) analysis. The grading criteria were defined as follows: 0, no visible leaf curling or chlorosis; 1, slight leaf curling and/or mild chlorosis; 2, clear leaf curling accompanied by obvious chlorosis; and 3, severe leaf curling, pronounced yellowing, and marked leaf deformation. The visual assessment was performed by two investigators independently before the ELISA and ddPCR results were available. This balanced panel was designed to provide a controlled baseline for evaluating assay response across symptom levels, rather than to represent population-level field epidemiology. To reduce confounding during this initial proof-of-concept evaluation, samples were pre-screened to exclude mixed infections with the tested heterologous RNA viruses. The sample set should therefore be interpreted as a preliminary field-sample panel, not as a fully representative field validation cohort. Accordingly, this panel was not used to derive formal estimates of diagnostic sensitivity, diagnostic specificity, false-positive or false-negative rates, predictive values, or routine operational performance.

### 2.8. ddPCR Absolute Quantification

The 32 field-collected tomato samples were analyzed in parallel by ELISA and ddPCR for preliminary orthogonal evaluation. Samples were analyzed by ELISA using the optimized dilution factor, whereas total genomic DNA was extracted for ddPCR quantification. ddPCR served as an orthogonal molecular reference for comparing ELISA absorbance values with TYLCV DNA concentrations. Droplet generation and amplification were performed using a DQ24 digital PCR system (Sniper Biosciences, Beijing, China) with a commercial 2 × T5 Fast qPCR Mix (Probe) (Tsingke Biotechnology, Beijing, China). Reaction conditions and primer-probe information are provided in Table S2. Absolute TYLCV DNA concentrations were calculated by the DQ24-Sight software (version 1.3.0, Sniper Medical Technologies Co., Ltd., Suzhou, China) from the numbers of positive and negative droplets using Poisson-based correction and were reported as copies/μL. A plasmid containing the TYLCV target sequence was included as the positive control and produced the expected positive droplet population, whereas a no-template control was included as the negative control and showed no positive droplets. Wells containing fewer than 10,000 accepted droplets were excluded. Linear regression analyses between ELISA absorbance and ddPCR-reported TYLCV DNA concentration, and between ELISA absorbance and DI value, were performed using Origin 2021 software (OriginLab Corporation, Northampton, MA, USA).

### 2.9. Preliminary Computational Structural Analysis of the Recombinant Antigen

To provide a preliminary computational reference for antigen interpretation, the monomeric structure of the TYLCV CP sequence used in this study (NCBI accession No. ABG73017.1) was predicted using AlphaFold2, version 2.3.2 (Google DeepMind, London, UK). A Trx-His-CP fusion model was also generated to examine the possible spatial arrangement between the retained Trx-His tag and the CP domain. Predicted solvent-exposed regions were visualized using PyMOL, version 3.1.8 (Schrödinger, LLC, New York, NY, USA) and interpreted only as computational features of the predicted model.

LC-MS/MS-detected peptide sequences were mapped onto the predicted CP model to visualize sequence coverage in structural context. This mapping was used to compare experimentally detected peptide coverage with predicted model topology. It was not interpreted as direct evidence of native surface exposure or antibody epitope localization. Pairwise CP sequence similarity between TYLCV and TbCSV was analyzed using the NCBI BLASTp web server (National Center for Biotechnology Information, Bethesda, MD, USA; accessed on 18 July 2026). Identical or highly similar residues were visualized on the predicted CP model as a preliminary reference for interpreting the observed cross-recognition. Because AlphaFold2 predicts a monomeric model rather than the assembled *geminivirus* particle, this analysis was used only as an interpretive reference and not as direct structural validation of the refolded antigen or native virion-surface conformation.

## 3. Results

### 3.1. Preparation, Refolding and Characterization of Recombinant TYLCV CP Antigen

**Figure 1:**
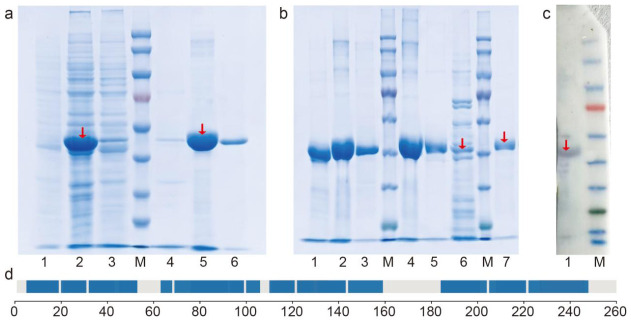
Expression, purification, and identification of the recombinant Trx-His-CP fusion protein. (**a**) SDS-PAGE analysis of induced expression and cellular disruption fractions. Lanes 1 and 2: whole bacterial cells before and after IPTG induction; Lanes 3 and 4: supernatant after cell disruption; Lanes 5 and 6: supernatant and pellet fractions after inclusion body solubilization; (**b**) Ni-NTA affinity chromatography and characterization of refolded products. Lane 1: pellet fraction after cell disruption; Lanes 2 and 3: supernatant and pellet fractions post-refolding; Lanes 4 and 5: flow-through and wash fractions; Lane 6: supernatant prior to denaturation and refolding; Lane 7: purified target protein derived from inclusion body pellets post-denaturation and refolding; (**c**) Specificity verification via Western blot analysis, where the refolded protein was recognized by an anti-His mAb. The red arrows in panels (**a**–**c**) indicate the recombinant Trx-His-CP fusion protein band at approximately 48 kDa; (**d**) LC-MS/MS sequence coverage map, where the grey area represents the full-length CP sequence (1–259 aa) and the blue area highlights the specific peptides detected by mass spectrometry (sequence coverage: 85.3%). M: protein molecular weight marker. The neighbouring bands for the target protein (approximately 48 kDa) correspond to 55 kDa and 40 kDa in (**a**), and to 60 kDa and 45 kDa in (**b**,**c**). Detailed standard band profiles for the markers are provided in Supplementary Figure S5a,b.

To obtain recombinant CP antigen for antibody preparation, the expression, purification and refolding of the target protein were systematically evaluated. Screening of induction conditions indicated that induction at 20 °C for 16 h produced the highest expression level of the recombinant Trx-His-CP fusion protein (Figure S3), with a distinct target band observed at approximately 48 kDa. Solubility analysis showed no clear target band in the supernatant after cell lysis under the tested induction conditions (Figure 1a), indicating predominant expression as inclusion bodies. The inclusion bodies were solubilized in 8 M urea and purified by Ni-NTA affinity chromatography. The eluate showed a single target band at 48 kDa (Figure 1b). After gradient dialysis at 0.3 mg/mL, 17 mg of refolded protein was recovered from 5 L fermentation culture. ImageJ densitometric analysis indicated an approximate purity of 98.8% (Figure S4), and Western blotting confirmed recognition by an anti-His monoclonal antibody (mAb) (Figure 1c). These results supported the integrity of the fusion tag and target protein sequence.

LC-MS/MS analysis was performed to further confirm antigen identity and evaluate host residual proteins. A total of 36 TYLCV CP-derived peptides were identified, with a total protein score of 323.31 and sequence coverage of 85.3% (Figure 1d and Figure S6; Table S3). The MS/MS fragmentation spectrum of the representative high-abundance peptide INSHVTYNHQEAAK showed a clear and continuous fragment ion series (Figure S7). For purity assessment, total composition abundance analysis indicated that the target fusion protein signal was 7.65 × 10^11^, nearly two orders of magnitude higher than that of the most abundant *E. coli* host residual protein (Table S4). For the 14.7% of sequence not detected by LC-MS/MS, physicochemical analysis suggested that trypsin cleavage patterns and high local densities of lysine and arginine residues may have generated short peptides outside the effective detection range of standard database searches. Therefore, LC-MS/MS coverage was used to support antigen identity and purity, rather than to infer native surface accessibility or antibody-recognition regions. Overall, the purified recombinant Trx-His-CP fusion antigen showed broad sequence coverage and low detectable host-protein contamination, supporting its use for antibody preparation.

### 3.2. pAb Preparation and Establishment of the ELISA System

**Figure 2:**
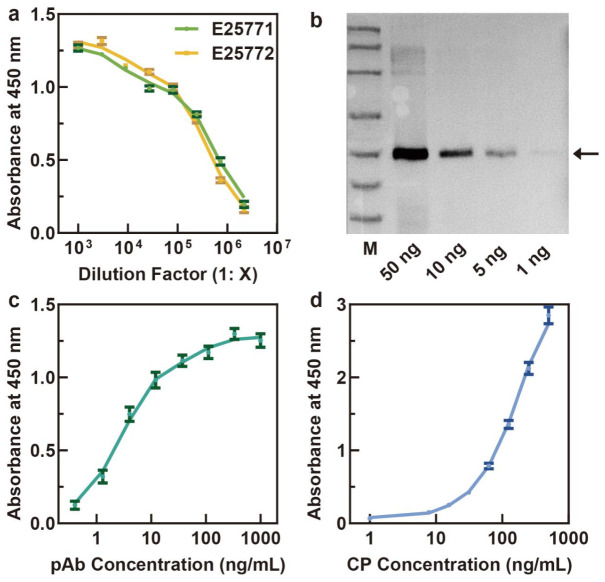
Preparation, identification, and ELISA system establishment of the pAb. (**a**) Antiserum titration curves of the immunized rabbits (E25771 and E25772); (**b**) Western blot evaluation of purified pAb recognition of serially loaded recombinant Trx-His-CP fusion antigen. Lanes from left to right represent the molecular-weight marker (M) and recombinant Trx-His-CP loaded at 50, 10, 5, and 1 ng. The arrow indicates the recombinant Trx-His-CP antigen at approximately 48 kDa. The neighbouring marker bands correspond to 70 and 40 kDa, and the detailed marker profile is provided in Supplementary Figure S5. No separate negative-control lane was included in this serial-loading experiment. Potential Trx-directed background reactivity was assessed separately by ELISA using recombinant Trx protein (Figure S8); (**c**) Titration of the purified pAb against a fixed antigen concentration (100 ng/mL); (**d**) Calibration standard curve of the developed ELISA (1–500 ng/mL). Data are expressed as mean ± SD (*n* = 3).

To generate a reactive antibody reagent for TYLCV detection, Japanese white rabbits were immunized with the purified recombinant Trx-His-CP fusion antigen. Antiserum titers from both rabbits were monitored by indirect ELISA (Figure 2a). Within the working dilution range of 1:1000 to 1:81,000, the antiserum from rabbit E25772 showed a more gradual signal decrease and a more stable response profile. Therefore, this antiserum was selected for antigen-affinity purification using the recombinant Trx-His-CP fusion antigen, yielding a purified pAb concentration of 1.03 mg/mL.

Western blot analysis using serially loaded recombinant Trx-His-CP fusion antigen showed that the antigen-affinity-purified pAb, used at a working concentration of 1 μg/mL, detected the expected antigen band at approximately 48 kDa, with a visible signal at antigen loads of 5 ng and above (Figure 2b). The detected band corresponded to the recombinant Trx-His-CP fusion antigen rather than to the purified pAb itself. These results demonstrate that the purified pAb recognized the recombinant fusion antigen under the tested Western blot conditions.

Optimal reaction conditions were determined by checkerboard titration (Table S5). The highest S/N value (9.8) was achieved with a CP antigen coating concentration of 100 ng/mL and a pAb working concentration of 10 ng/mL. Under these conditions, the pAb titration curve showed a sigmoidal profile, with a half-maximal effective concentration (EC_50_titration_) of 3.09 ng/mL (Figure 2c). The calibration curve showed a good fit to the 4PL model across the tested concentration range of 1–500 ng/mL (*R*^2^ = 0.999, Figure 2d), yielding a theoretical LOD of 1.8 ng/mL. Together, these parameters indicate that the optimized detection system provides high sensitivity across a broad quantitative range.

Because both immunization and affinity purification used the recombinant Trx-His-CP fusion antigen, potential tag-directed background reactivity was evaluated using recombinant Trx control protein and BSA as control antigens (Figure S8). Across the tested pAb dilution range, the response to Trx control protein remained substantially lower than the response to target Trx-His-CP fusion antigen. At the highest pAb concentration tested (1:1000 dilution), the Trx control signal reached an OD_450_ value of 0.203, below the positive cut-off threshold of 0.24. However, the margin was narrow. These results suggest that diagnostic reactivity was mainly associated with the TYLCV CP region under the tested conditions, although low-level tag-directed background reactivity cannot be excluded.

### 3.3. Analytical Performance and Preliminary Field-Sample Evaluation of the Indirect ELISA

**Figure 3:**
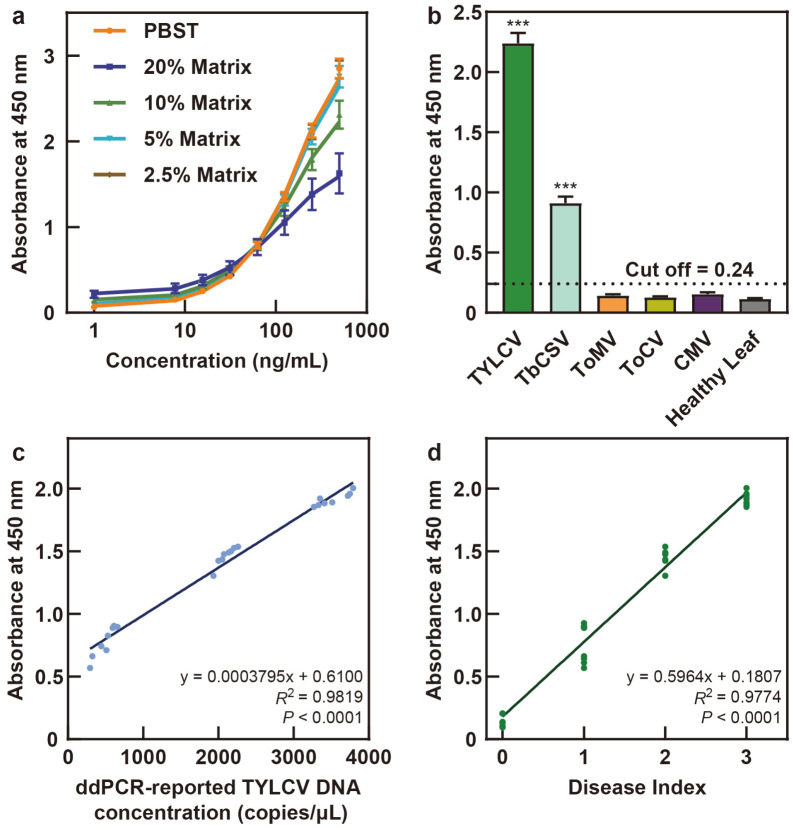
Analytical performance and preliminary field-sample evaluation of the developed indirect ELISA. (**a**) Matrix effects of different tomato extract concentrations on the detection system; (**b**) Cross-reactivity profile against a closely related *begomovirus*, heterologous RNA viruses, and healthy plant matrix; (**c**) Linear regression analysis between ELISA absorbance and ddPCR-reported TYLCV DNA concentration within the tested sample panel; (**d**) Association between ELISA absorbance and DI score within the tested sample panel. Data are presented as mean ± SD (n = X). *** *p* < 0.001 versus the healthy leaf control by one-way ANOVA followed by Dunnett’s test.

To minimize background interference from tomato leaf metabolites such as polyphenols, matrix effects were evaluated by serially diluting healthy leaf homogenates. A 20.0% homogenate concentration caused 45.2% signal inhibition, whereas dilution to 2.5% reduced the matrix effect to −1.2% and restored favourable linearity to the standard curve (*R*^2^ = 0.9985; Table 1 and Figure 3a). At this optimized dilution, spike recoveries ranged from 95.45% to 100.40%, and both intra-assay and inter-assay CVs remained below 5.0% (Table 2; Tables S6 and S7). The recovery value slightly above 100% likely reflects minor analytical variation or weak matrix-related signal enhancement and should be interpreted as near-complete recovery rather than excess antigen recovery. These results indicate that 2.5% sample dilution minimized matrix interference and supported assay accuracy and reproducibility.

To further characterize specificity, cross-reactivity against a closely related congeneric virus and common heterologous pathogens was evaluated using infected samples at the 2.5% dilution factor. The assay produced a strong recognition signal for TYLCV and a moderate cross-reactive signal for the related *begomovirus* TbCSV, whereas no cross-reactivity was observed against the heterologous RNA viruses ToMV, ToCV or CMV (Figure 3b). These findings indicate favourable discrimination against the tested unrelated RNA viruses. However, the positive response to TbCSV indicates that the assay cannot distinguish TYLCV from TbCSV and should not be considered strictly TYLCV species-specific.

To evaluate the assay in a preliminary field-sample panel, the pre-screened balanced cohort was analyzed by parallel ELISA, ddPCR and DI assessment. Qualitative ELISA classifications corresponded to the ddPCR results for all samples within this selected panel (Table S8). However, because the panel was deliberately balanced by symptom severity and pre-screened before inclusion, this descriptive agreement was not used to estimate diagnostic sensitivity, diagnostic specificity, false-positive or false-negative rates, or routine operational performance. In quantitative regression analysis, ELISA absorbance showed a significant positive association with ddPCR-reported TYLCV DNA concentrations within the tested panel (*R*^2^ = 0.9819, *p* < 0.001, Figure 3c). Considering the 40-fold dilution factor required to reduce matrix interference (2.5% sample matrix), the theoretical LOD of 1.8 ng/mL corresponded to a practical detectable concentration of 72 ng/mL under the optimized dilution condition. A distinct colorimetric response was observed even in mild-symptom samples with ddPCR-reported TYLCV DNA concentrations ranging from 296.29 to 659.93 copies/μL.

ELISA absorbance also showed a strong linear correlation with plant DI values (*R*^2^ = 0.9774, *p* < 0.001; Figure 3d). Overall, the ELISA response varied with ddPCR-reported TYLCV DNA concentration and visual symptom severity across the ranges represented in this selected sample panel. These findings support the preliminary evaluation of assay responsiveness in the tested field-collected samples but do not constitute formal diagnostic-performance validation.

### 3.4. Preliminary Analysis of Antigen Structural Features and Experimental Data Mapping

**Figure 4:**
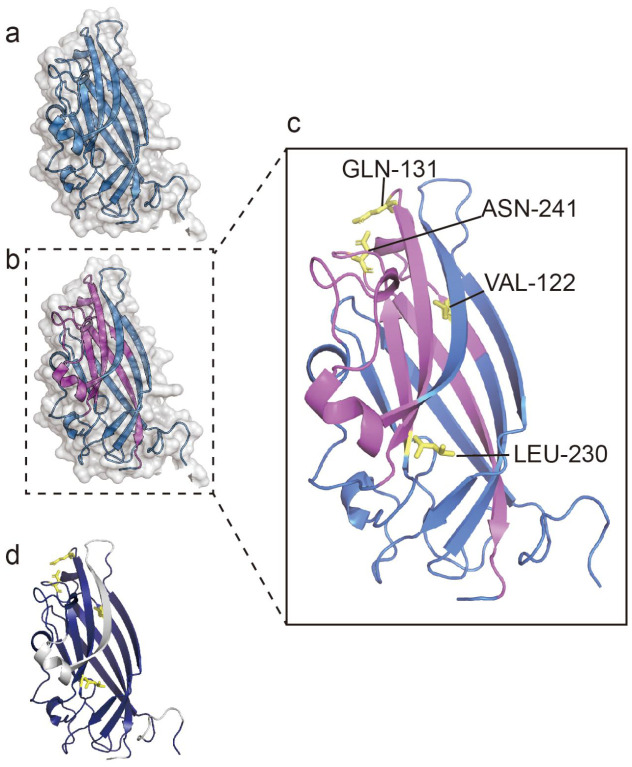
Preliminary computational visualization of the TYLCV CP model and LC-MS/MS peptide coverage. (**a**) AlphaFold2-predicted monomeric TYLCV CP model, with the secondary structure skeleton shown in blue and the translucent molecular surface shown in grey. (**b**) Putative solvent-exposed features visualized on the predicted monomeric model, with magenta regions indicating predicted outer-surface regions. (**c**) Local visualization of representative surface-distributed residues, including Gln131, Val122, Asn241, and Leu230. These residues are shown for spatial visualization and do not represent experimentally validated antibody-recognition sites. (**d**) LC-MS/MS-detected peptides mapped onto the AlphaFold2-predicted monomeric TYLCV CP model to visualize sequence coverage in a structural context. LC–MS/MS-detected peptide regions are shown in white, whereas the remaining CP structure is shown in dark blue; the selected representative residues are shown as yellow sticks. This mapping does not represent experimental determination of native surface exposure or antibody epitopes.

To provide a preliminary computational context for interpreting the recombinant antigen, an AlphaFold2-predicted monomeric model of the TYLCV CP sequence was generated (Figure 4a). The pLDDT profile indicated generally favourable prediction confidence across the core region, whereas lower-confidence segments may represent flexible regions and should be interpreted cautiously (Figure S9). Because this model represents a predicted monomeric CP structure rather than the assembled geminivirus particle, it was used only as a computational reference.

Predicted solvent-exposed features were visualized using PyMOL (Figure 4b). These regions were interpreted as putative surface features of the predicted monomeric model, not as experimentally determined native virion-surface regions. Representative surface-distributed residues, including Gln131, Val122, Asn241 and Leu230, were selected for local visualization (Figure 4c). These residues were used to illustrate spatial distribution on the predicted model and should not be interpreted as experimentally validated antibody-recognition sites.

To place the LC-MS/MS data in a structural context, the detected peptide sequences were mapped onto the AlphaFold2-predicted monomeric CP model (Figure 4d). The mapped peptides showed broad distribution across the predicted CP structure, consistent with the broad LC-MS/MS sequence coverage. However, this mapping should not be interpreted as experimental evidence of native surface exposure or antibody epitope localization, because peptide detection is influenced by trypsin cleavage patterns, peptide length, hydrophobicity, and ionization efficiency. Therefore, the peptide mapping was used only to visualize sequence coverage in a computational structural context.

To provide a preliminary sequence-level context for the observed cross-recognition toward TbCSV, TYLCV and TbCSV CP sequences were compared using BLASTp. Pairwise comparison between TYLCV CP (ABG73017.1) and TbCSV CP (CAD12412.1) showed approximately 78% amino acid identity. This similarity may partly explain the observed cross-recognition toward TbCSV. However, the TYLCV-TbCSV CP comparison does not define antibody epitopes or native virion-surface accessibility.

## 4. Discussion

### 4.1. Preparation and Characterization of the Recombinant Trx-His-CP Antigen

In this study, an MCS-free direct-fusion construct was used to produce a recombinant TYLCV Trx-His-CP fusion antigen, followed by gradient refolding. The resulting preparation showed high purity, broad LC-MS/MS sequence coverage, and limited detectable host-protein contamination, and it successfully supported pAb generation and subsequent ELISA development. These findings demonstrate the feasibility of the antigen-production workflow under the tested conditions. However, because no MCS-retaining or linker-containing construct, tag-cleaved antigen, or tag-free CP control was included, the present data do not establish that removal of the MCS-derived intermediate residues improved protein folding, epitope accessibility, immunoreactivity, or assay performance.

In heterologous expression systems, MCS-derived intermediate sequences or fusion-tag-associated residues may be present between a target protein and a fusion carrier [15,18]. Such sequence features have been discussed as potential factors affecting recombinant antigen presentation in some expression systems; however, their specific effects on the present TYLCV Trx-His-CP antigen were not directly evaluated. The retained Trx-His tag was further considered because tag-directed antibody responses may contribute to background reactivity in fusion-antigen-based immunoassays. In the present study, the pAb showed strong reactivity toward the recombinant Trx-His-CP fusion antigen, whereas its binding to the recombinant Trx control protein remained below the positive threshold across the tested dilution range (Figure S8). This result indicates limited detectable tag-directed background reactivity under the tested assay conditions. Nevertheless, it does not demonstrate the complete absence of anti-tag antibodies.

A further limitation concerns the antibody purification strategy. In this study, rabbits were immunized with the recombinant Trx-His-CP fusion antigen, and the same fusion antigen was used for affinity purification. Therefore, the purified pAb preparation should be interpreted as predominantly CP-reactive rather than completely tag-depleted. Although the Trx control signal remained below the positive cut-off threshold across the tested dilution range, low-level tag-directed background reactivity cannot be completely excluded. Future work should include Trx-based negative depletion, tag-cleaved CP controls, or tag-free antigen controls to further reduce and characterize tag-directed background reactivity.

Despite the favourable immunological outcomes, several limitations remain. A direct experimental comparison with a conventional linker-containing construct, tag-cleaved antigen or tag-free CP control was not performed. The contribution of MCS removal to epitope accessibility and immunoreactivity should therefore be interpreted as a design rationale rather than a directly demonstrated mechanism. In addition, recombinant antigen surface features were evaluated only indirectly, through correspondence between LC-MS/MS peptide mapping and the AlphaFold2 prediction. Secondary structure composition and oligomeric status were not examined by circular dichroism, size-exclusion chromatography, dynamic light scattering or native PAGE. Consequently, the observed ELISA performance supports the suitability of the prepared antigen for ELISA development under the tested conditions, but does not establish the superiority of the MCS-free construct over alternative antigen designs.

It should also be noted that the recombinant antigen used in this study was obtained from inclusion bodies after urea solubilization and gradient dialysis. Although SDS-PAGE, Western blotting, and LC-MS/MS supported the purity and sequence identity of the refolded Trx-His-CP fusion antigen, these analyses do not demonstrate that the recombinant antigen fully reproduces the conformational state of CP on native virions. In native geminivirus particles, CP participates in higher-order assembly and interacts with viral DNA, whereas the recombinant antigen prepared here represents a refolded fusion protein. Therefore, the antibody response generated in this study may include antibodies against linear, partially exposed, or refolding-dependent epitopes rather than exclusively native virion-surface conformational epitopes. Future studies should use complementary biophysical and structural approaches, such as circular dichroism, size-exclusion chromatography, dynamic light scattering, native PAGE, and, where feasible, cryo-electron microscopy or X-ray crystallography, to further characterize the folding state, oligomeric status, and antibody-recognition features of the recombinant antigen [19,20].

### 4.2. Matrix Tolerance, Cross-Reactivity, and Preliminary Field-Sample Evaluation of the Indirect ELISA

The developed indirect ELISA showed strong positive associations with ddPCR-reported TYLCV DNA concentrations and symptom-severity scores within the tested panel. A 2.5% sample dilution reduced the matrix effect to −1.2%. Under this optimized dilution condition, the assay detected TYLCV-positive samples and showed cross-recognition toward TbCSV, while no detectable cross-reactivity was observed with the tested heterologous RNA viruses. With an estimated pre-dilution equivalent LOD of 72 ng/mL after sample dilution, the assay enabled preliminary evaluation of field-collected samples across symptom-severity levels. These results support the analytical matrix tolerance of the assay under the tested conditions.

Under field conditions, secondary metabolites such as polyphenols in plant sap can contribute to background interference, and viral co-infections may further complicate serological interpretation [21,22]. The sample dilution strategy changed the theoretical LOD of 1.8 ng/mL into a practical threshold of 72 ng/mL. In this pilot panel, ELISA signals remained detectable in low-viral-load samples. This observation may reflect the accumulation of CP-associated antigenic determinants during active viral replication [23,24]. However, it should be interpreted within the limited sample set tested here, not as evidence of broad field diagnostic sensitivity. In addition, ddPCR quantifies viral DNA, whereas ELISA detects CP-associated antigenic determinants. The observed correlation between ELISA absorbance and ddPCR-reported TYLCV DNA concentration should therefore be interpreted as an empirical association within the tested samples, rather than a fixed quantitative relationship between genome copies and CP antigen abundance. Accordingly, the practical advantage of the assay lies mainly in its low-cost, high-throughput screening workflow rather than in species-level molecular confirmation.

The cross-recognition toward TbCSV has direct implications for diagnostic interpretation. A TbCSV single infection may generate a positive ELISA signal that could be misclassified as TYLCV, whereas the assay cannot distinguish the relative contributions of TYLCV and TbCSV in a mixed infection. Therefore, the current method should be regarded as a preliminary serological screening assay rather than a strictly TYLCV species-specific diagnostic test. Species-specific PCR, ddPCR, or sequencing remains necessary when related *begomoviruses* may be present or when species-level identification is required.

This cross-recognition may be partly associated with CP sequence conservation among related *begomoviruses* [25,26]. TbCSV has been reported infecting pepper in Sichuan Province, China [27]. However, published quantitative information on the prevalence of TbCSV in tomato production systems in Beijing, where the samples in the present study were collected, remains limited. Because the present balanced 32-sample panel was designed for preliminary methodological evaluation rather than regional epidemiological surveillance, the local frequency of TbCSV and the operational false-positive risk associated with cross-recognition cannot be estimated from the current dataset. Region-specific surveillance using species-specific molecular assays, together with testing against a broader panel of tomato-infecting *begomoviruses*, will be required before routine deployment of this assay.

Healthy leaf extracts produced baseline background signals ranging from 0.0946 to 0.2071 at OD_450_. These background signals may have originated from trace nonspecific adsorption or minor substrate autoxidation [28,29]. Based on these background values, the positive cut-off threshold was defined as 0.24. All healthy samples remained below this threshold and were classified as negative under the tested assay conditions. Direct head-to-head comparison with commercial or previously reported TYLCV ELISA kits was not performed; therefore, the present assay should not be interpreted as superior to existing serological methods.

Previous TYLCV serological assays have differed substantially in antibody type, assay format, analytical sensitivity, specificity, and validation scope. Wu et al. developed murine mAbs against recombinant TYLCV CP and established a TAS-ELISA that detected infected plant sap diluted up to 1:2560. However, the mAb used to establish that assay recognized five tomato-infecting *begomoviruses*, indicating broad *begomovirus* reactivity rather than strict TYLCV species specificity [30]. Xie et al. subsequently used purified TYLCV virions to generate mAbs and developed mAb 1C4-based dot-ELISA and DTBIA. Their dot-ELISA detected infected tomato extract diluted up to 1:5120 and viruliferous whitefly homogenate diluted up to 1:128. The methods were evaluated using 487 field tomato samples and 110 whitefly samples collected from three provinces, with PCR and nucleotide sequencing used for confirmation; however, mAb 1C4 also recognized multiple *begomoviruses*, including TbCSV [31]. Earlier work showed that indirect plate-trapping ELISA detected TYLCV in purified preparations but was ineffective in crude plant extracts, whereas chemiluminescent dot-ELISA provided improved detection in stem-squash samples [32].

In comparison, the present study used a rabbit pAb raised against recombinant Trx-His-CP in an indirect microplate ELISA. The assay showed an analytical LOD of 1.8 ng/mL against recombinant antigen and was preliminarily evaluated using a deliberately balanced panel of 32 field-collected tomato samples, with ddPCR used as an orthogonal molecular reference. No detectable reactivity was observed against the tested unrelated RNA viruses, whereas cross-recognition of TbCSV was detected, indicating partial discrimination but not strict TYLCV species specificity. Because the previous studies and the present assay used different antibody types, antigen sources, assay formats, sample matrices, sensitivity endpoints, and validation designs, their reported sensitivity values cannot be compared directly. The present assay should therefore be regarded as an alternative laboratory approach rather than a demonstrated practical improvement over established TYLCV serological methods. Direct benchmarking against established or commercially available assays will be required to determine comparative practical performance.

The field-sample analysis should be interpreted as a preliminary methodological evaluation rather than as population-level diagnostic validation. The 32 samples were intentionally assembled as a balanced and pre-screened panel across four symptom-severity categories and were collected from Beijing, China, during the winter of 2025 and the spring of 2026. This design allowed the associations between ELISA absorbance, ddPCR-reported TYLCV DNA concentration, and DI score to be examined within the tested panel. However, because the panel was deliberately balanced by symptom severity, geographically restricted, and pre-screened before inclusion, the observed correspondence between qualitative ELISA classifications and ddPCR results, as well as the quantitative associations, cannot be used to estimate diagnostic sensitivity, diagnostic specificity, false-positive or false-negative rates, predictive values, or routine operational performance. The panel also does not fully reflect the complexity of operational field settings, in which mixed infections, cultivar differences, seasonal variation, and atypical or asymptomatic infections may occur. Moreover, formal receiver operating characteristic (ROC) analysis was not performed. Formal diagnostic evaluation will therefore require larger, blinded, independently or consecutively collected, geographically and seasonally diverse cohorts containing both symptomatic and asymptomatic samples, followed by ROC analysis and estimation of diagnostic-performance parameters. Additional validation should include sequence-characterized TYLCV isolates and broader *begomovirus* panels. Operational factors relevant to field deployment, including reagent stability, inter-operator variability, workflow duration, portable-reader compatibility, and temperature robustness, were not evaluated and should be addressed in future work.

### 4.3. Preliminary Computational Context for Antigen Interpretation and Cross-Recognition

The AlphaFold2-predicted monomeric CP model and LC-MS/MS peptide mapping were used only to provide a preliminary computational context for antigen interpretation. Putative solvent-exposed features were visualized on the predicted monomeric model, and LC-MS/MS-detected peptides were mapped onto this model to show sequence coverage in a structural context. However, this mapping does not establish native virion-surface exposure, antibody epitopes, or antigen–antibody complex structure.

Previous studies suggest that fusion tags or misfolding may influence antigen presentation by affecting the accessibility of some antigenic regions [15,18]. In the present study, the MCS-free Trx-His-CP fusion antigen showed favourable immunoreactivity in ELISA, but the contribution of the predicted surface features to antibody binding was not directly demonstrated. The exact epitopes recognized by the prepared pAb therefore remain undefined. In addition, *geminivirus* CPs show sequence conservation within assembly-related domains [33,34], which may partly contribute to the observed cross-recognition toward TbCSV. Nevertheless, the analysis of cross-recognition was restricted to one related *begomovirus*, and broader testing is required to define the cross-reactivity spectrum.

Taken together, the computational structural analysis should be interpreted as an auxiliary reference rather than mechanistic validation. The potential operational value of the assay lies in its relatively simple, high-throughput screening format; however, cost, workflow duration, and routine operational performance were not directly benchmarked in this study. The assay demonstrated preliminary detection capability in the tested TYLCV-positive field samples. However, the intraspecies recognition breadth of the assay was not specifically evaluated because the field samples were not characterized at the isolate level. Future work should prioritize larger field validation, evaluation using geographically and genetically diverse, sequence-characterized TYLCV isolates, broader *begomovirus* panels, and, where feasible, experimental structural or epitope-mapping approaches.

## 5. Conclusions

This study supports the feasibility of producing a recombinant Trx-His-CP antigen, generating a rabbit pAb, and establishing an indirect ELISA for preliminary serological screening of tomato samples. The antigen-production and assay-development workflow functioned under the tested conditions; however, the present findings do not establish that the MCS-free construct is superior to alternative antigen configurations. The assay responses were associated with ddPCR-reported TYLCV DNA concentrations and symptom-severity scores within the tested panel, but cross-recognition of TbCSV indicates that the assay is not strictly TYLCV species-specific.

Because the study used a deliberately balanced panel of 32 samples from one geographical area and two sampling periods, it does not provide formal estimates of diagnostic sensitivity, specificity, predictive values, false-positive or false-negative rates, or routine operational performance. In the absence of direct benchmarking against established TYLCV serological assays, the method should be regarded as an alternative laboratory screening approach rather than a demonstrated practical improvement. Further development should include direct comparative testing, blinded multi-region evaluation using larger independently or consecutively collected symptomatic and asymptomatic cohorts, ROC-based threshold optimization, evaluation of sequence-characterized TYLCV isolates and broader *begomovirus* panels, and assessment of reagent stability, inter-operator variability, workflow duration, portable-reader compatibility, and temperature robustness.

## Figures and Tables

**Table 1:** Matrix effect and curve-fitting parameters of the ELISA in varying concentrations of tomato leaf extract.

Matrix Concentration (%)	*A_max_* (OD_450_)	EC_50_ (ng/mL)	*R* ^2^	ME (%)
PBST (Standard)	3.052 ± 0.124	148.5	0.9992	0.0%
2.5% Matrix	2.985 ± 0.105	150.2	0.9985	−1.2%
5.0% Matrix	2.854 ± 0.118	158.4	0.9976	−5.8%
10.0% Matrix	2.421 ± 0.142	182.1	0.9965	−21.5%
20.0% Matrix	1.752 ± 0.215	142.6 *	0.9928	−45.2%

Note: PBST, PBS containing 0.05% tween-20; *A_max_,* fitted maximum absorbance value; EC_50_, half-maximum binding concentration; *R*^2^, coefficient of determination; ME (%) = [(Slope _matrix_/Slope _PBST_) − 1] × 100%. * The apparent decrease in EC_50_ is attributed to the marked inhibition of the maximum signal plateau by high matrix concentrations.

**Table 2:** Recovery and precision of the ELISA for TYLCV CP spiked in a 2.5% tomato leaf matrix.

Spiked CP (ng/mL)	Measured CP (ng/mL) ^a^	Recovery (%) ^b^	Intra-Assay CV (%)	Inter-Assay CV (%) ^c^
25.00	23.90 ± 0.28	95.60	1.16	4.91
100.00	100.40 ± 1.97	100.40	1.96	3.81
400.00	381.83 ± 6.53	95.45	1.71	3.56

Note: ^a^ Data are expressed as mean ± SD (*n* = 3); ^b^ Recovery rate (%) = (mean detected concentration/spiked concentration) × 100%; ^c^ Calculated from experimental data of three independent batches conducted on separate days. Values slightly above 100% reflect minor analytical variation and possible matrix-related signal enhancement.

## Data Availability

Data are contained within the article and Supplementary Materials.

## Supplementary Materials

The following supporting information can be downloaded at: https://www.mdpi.com/article/10.3390/v18070786/s1, Figure S1: Schematic illustration of the MCS-free direct-fusion construct used for recombinant Trx-His-CP antigen production. Figure S2: Immunization protocol and preparation process of the specific pAb; Figure S3: Optimization of induction conditions and solubility analysis of the recombinant CP fusion protein; Figure S4: Densitometric purity analysis of the refolded recombinant protein using ImageJ software; Figure S5: Detailed standard band profiles for the protein molecular weight markers; Figure S6: LC-MS/MS sequence coverage map of the recombinant antigen; Figure S7: MS/MS fragmentation spectrum of the representative high-abundance peptide INSHVTYNHQEAAK; Figure S8: Specificity control test and pAb response curves against the recombinant Trx control protein; Figure S9: Per-residue model confidence scores (pLDDT) plot for the predicted TYLCV CP structure; Table S1: Primers used for the construction of the recombinant expression vector pET32a-CP; Table S2: Primers used for ddPCR; Table S3: Specific peptides identified by LC-MS/MS; Table S4: Total composition abundance analysis of the purified antigen; Table S5: Optimization of ELISA reaction parameters by checkerboard titration; Table S6: Raw data for analytical recovery and intra-assay precision evaluation of TYLCV CP spiked into healthy tomato leaf extracts (2.5% matrix); Table S7: Raw data for inter-assay precision evaluation of TYLCV CP protein spiked into healthy tomato leaf extracts (2.5% matrix) across independent test plates; Table S8: Individual diagnostic results of 32 field tomato samples.

## Abbreviations

The following abbreviations are used in this manuscript:

TYLCV	Tomato yellow leaf curl virus
CP	Coat protein
pAb	Polyclonal antibody
ELISA	Enzyme-linked immunosorbent assay
ddPCR	Droplet digital PCR
LC-MS/MS	Liquid chromatography-tandem mass spectrometry
OFAT	One-factor-at-a-time
DI	Disease index
