# Supplementary material for: Development and Preliminary Field Evaluation of an Indirect ELISA for Detecting Tomato Yellow Leaf Curl Virus
Source: Viruses. 2026 Jul 19;18(7):786. doi: 10.3390/v18070786 (PMC13431607; doi:10.3390/v18070786)
Supplement: Supplementary file 1 [file viruses-18-00786-s001.zip › viruses-4409781-supplementary.pdf]

Table S1. Primers used for the construction of the recombinant expression vector pET32a-CP.

Primer Name	Sequence (5'to 3')	Description / Homology Arm
CP-F	gctgatatcggatccGAATTCATGGCGAGCAAACGTC	Homology to pET32a (*BamH*I)
CP-R	gtggtggtggtggtgCTCGAGATTGCTGATGCTATCGTAGAAA	Homology to pET32a (*Xho*I)

Note:

Homology arms are shown in lowercase letters. Sequences complementary to the target gene (TYLCV CP) are shown in uppercase letters. The restriction enzyme sites (*BamH*I and *Xho*I) are embedded within the homology arms.

Table S2. Primers used for ddPCR.

Primer Name	Sequence (5' to 3')	Description
TYLCV-F	TTCGCCGAAGGCTGAACTTC	Forward primer
TYLCV-R	CCTGTACGTCCATGATCGTCG	Reverse primer
TYLCV-P	FAM-TGTTTGTGCCTTGGACAATGGGGAC-BHQ1	TaqMan probe

Note:

TYLCV-F and TYLCV-R target the CP gene of TYLCV. TYLCV-P is the TaqMan probe labeled with FAM at the 5'end and BHQ1 at the 3' end. Detailed ddPCR reaction conditions are described in Section 2.8.

Table S3. Specific peptides identified by LC-MS/MS.

No.	Peptide Sequence	Length	Start-End	Score	Mass (Da)	Missed Cleavages
1	INSHVTYNHQEAAK	14	208-221	244.26	1610.78	0
2	KQNHTNQVMFFLVR	14	130-143	199.09	1760.91	1
3	FCVKSIYFLGK	11	111-121	179.1	1360.72	1
4	VQSYEQR	7	76-82	162.07	908.44	0
5	AAVPIVQGTNK	11	32-42	155.07	1096.62	0
6	LNFDSPYSSR	10	22-31	153.08	1184.55	0
7	RLNFDSPYSSR	11	21-31	150.35	1340.65	1
8	VQSYEQRDDIK	11	76-86	141.88	1379.67	1
9	GSGITHR	7	100-106	138.85	726.38	0
10	SPDVPR	6	63-68	137.5	669.34	0
11	SWTYRPMYR	9	45-53	135.83	1258.59	1
12	PGDIIISTPVSK	12	161-172	133.23	1225.69	0
13	DDIKHTGIVR	10	83-92	126.24	1152.63	1
14	EQALVKR	7	198-204	125.08	842.5	1
15	CVSDVTR	7	93-99	125.08	835.39	0
16	AAVPIVQGTNKR	12	32-43	116.37	1252.73	1
17	AAVPIVQGTNKRR	13	32-44	71.09	1408.83	2
18	DRRPYGNSPMDFGQVF	16	144-159	94.69	3319.5	2
19	FFRINSHVTYNHQEAAK	17	205-221	111.86	2061.02	1
20	FHATVIGGPSGMK	13	185-197	114.24	1300.66	0
21	FHATVIGGPSGMKEQAL	17	185-201	65.5	1969.05	1
22	GCEGPCKVQSYEQR	14	69-82	138.85	1696.73	1

No.	Peptide Sequence	Length	Start-End	Score	Mass (Da)	Missed Cleavages
23	HTGIVRCVSDVTR	13	87-99	112.42	1498.77	1
24	KFHATVIGGPSGMK	14	184-197	95.09	1428.75	1
25	KFHATVIGGPSGMKEQA	17	184-200	33.83	2097.14	2
26	PGDIIISTPVSKVR	14	161-174	153.08	1480.86	1
27	QNHTNQVMFFLVR	13	131-143	106.26	1632.82	0
28	RFCVKSIYFLGK	12	110-121	61.65	1516.82	2
29	RPGDIIISTPVSK	13	160-172	150.35	1381.79	1
30	RPGDIIISTPVSKVR	15	160-174	109.24	1636.96	2
31	RRLNFDSPYSSR	12	20-31	78.71	1496.75	2
32	RSWTYRPMYR	10	44-53	18.34	1414.69	2
33	VQSYEQRDDIKHTGIVR	17	76-92	105.2	2043.05	2
34	VWMDENIK	8	122-129	135.83	1033.49	0
35	VWMDENIKK	9	122-130	162.07	1161.59	1
36	YENHTENALLLYMACTH	17	232-248	141.88	3141.44	0

A total of 36 high-confidence peptides were detected, achieving an overall sequence coverage of 85.3%. The dataset outlines the amino acid sequence, residue position, and MaxQuant score for each identified peptide segment. The extensive distribution of these segments supports the sequence integrity of the prepared recombinant antigen.

Note:

Residue positions are numbered according to the full-length TYLCV CP sequence. Protein and peptide identifications were accepted at a false discovery rate (*FDR*) below 1%.

Table S4. Total composition abundance analysis of the purified antigen.

Rank	Protein Name / Identity	Species	Coverage (%)	Score	Intensity
1	Recombinant TYLCV-CP fusion protein	*Synthetic construct*	85.30 %	323.31	7.65×10^{11}
2	Thioredoxin (Trx fusion tag)	*Synthetic construct*	82.60 %	323.31	2.28×10^{11}
3	Efflux transporter, RND family, MFP subunit	*E. coli*	2.80 %	5.76	8.03×10^{9}
4	Tail assembly chaperone gp38	*E. coli*	6.50 %	5.82	2.29×10^{9}
5	Replication-associated recombination protein A	*E. coli*	2.00 %	5.8	2.00×10^{9}
6	Phosphoenolpyruvate synthase	*E. coli*	1.30 %	6.23	1.98×10^{9}
7	Asparagine--tRNA ligase	*E. coli*	11.40 %	40.08	1.26×10^{9}
8	ATP synthase subunit beta	*E. coli*	17.20 %	69.52	1.21×10^{9}
9	Surface-exposed outer membrane lipoprotein YaiW	*E. coli*	2.70 %	5.83	1.20×10^{9}
10	Transcription termination factor Rho	*E. coli*	22.40 %	131.13	7.45×10^{8}

Note:

The protein composition of the purified linker-free fusion antigen was analyzed via LC-MS/MS, with components ranked by signal intensity. The dataset outlines the top10 proteins, their species of origin, sequence coverage, and MS intensity. The signal intensity of the target fusion protein was approximately two orders of magnitude higher than the most abundant *E. coli* host contaminant (10^{11} vs 10^{9}). These data support the high purity of the prepared recombinant

Trx-His-CP fusion antigen without redundant intermediate linker sequences.

Table S5. Optimization of ELISA reaction parameters by checkerboard titration.

pAb Concentration (ng/mL)	Antigen: 50 ng/mL	Antigen:100 ng/mL	Antigen: 200 ng/mL
5	4.2	5.8	6.2
10	6.5	9.8*	9.2
20	6.8	8.5	8.1

Note:

The values represent the mean signal-to-noise (*S/N*) ratios derived from three independent technical replicates. The asterisk (*) indicates the optimal condition (100 ng/mL antigen and 10 ng/mL pAb) that achieved the highest S/N ratio of 9.8.

Table S6. Raw data for analytical recovery and intra-assay precision evaluation of TYLCV CP protein spiked into healthy tomato leaf extracts (2.5% matrix).

Spiked CP (ng/mL)	Measured CP (ng/mL)	Recovery (%)	Mean Recovery(%)	Intra-assay CV (%)
25.00	23.85	95.40		
25.00	24.20	96.80	95.60±1.25	1.16
25.00	23.65	94.60		
100.00	102.50	102.50		
100.00	98.60	98.60	100.40±2.05	1.96
100.00	100.10	100.10		
400.00	375.00	93.75		
400.00	388.00	97.00	95.45±1.62	1.71
400.00	382.50	95.63		

Note:

Individual measurements and calculations for analytical recovery and intra-assay precision evaluation. Recombinant TYLCV CP protein was spiked into healthy tomato leaf extracts at a 2.5% matrix concentration. Values slightly above 100% reflect minor analytical variation and possible matrix-related signal enhancement.

Table S7. Raw data for inter-assay precision evaluation of TYLCV CP protein spiked into healthy tomato leaf extracts (2.5% matrix) across independent test plates.

Spiked CP (ng/mL)	Test Batch	Recovery(%)	Overall Mean ± SD (ng/mL)	Inter-assay CV (%)
25.00	Plate 1 (Day 1)	95.40		
25.00	Plate 2 (Day 2)	96.80	23.95 ± 1.18	4.91
25.00	Plate 3 (Day 3)	94.60		
100.00	Plate 1 (Day 1)	102.50		
100.00	Plate 2 (Day 2)	98.60	100.13 ± 3.81	3.81
100.00	Plate 3 (Day 3)	100.10		

Spiked CP (ng/mL)	Test Batch	Recovery(%)	Overall Mean ± SD (ng/mL)	Inter-assay CV (%)
400.00	Plate 1 (Day 1)	93.75		
400.00	Plate 2 (Day 2)	97.00	381.81 ± 13.60	3.56
400.00	Plate 3 (Day 3)	95.63		

Note:

Data derived from three independent experimental batches conducted on separate days to assess inter-assay variation.

Table S8. Individual diagnostic results of 32 field tomato samples.

Sample ID	Symptom Grade	ddPCR Value (copies/µL)	ELISA OD_{450}	Qualitative Diagnosis
S1	3	3332.3588	1.8686	Positive
S2	3	3746.8342	1.958	Positive
S3	3	3515.2921	1.8904	Positive
S4	3	3412.9429	1.8838	Positive
S5	3	3790.1454	2.004	Positive
S6	3	3722.4284	1.9429	Positive
S7	3	3272.1856	1.8558	Positive
S8	3	3350.9011	1.92	Positive
S9	2	2051.7984	1.435	Positive
S10	2	2003.2824	1.4255	Positive
S11	2	2075.5873	1.476	Positive
S12	2	1935.1033	1.3055	Positive
S13	2	2139.6941	1.4906	Positive
S14	2	2167.9451	1.5003	Positive
S15	2	2208.4752	1.5263	Positive
S16	2	2260.1531	1.537	Positive
S17	1	296.2929	0.5685	Positive
S18	1	325.1246	0.6628	Positive
S19	1	445.8007	0.7424	Positive
S20	1	511.6334	0.7114	Positive
S21	1	531.644	0.8271	Positive
S22	1	595.1349	0.8894	Positive
S23	1	659.9304	0.8961	Positive
S24	1	610.6706	0.9044	Positive
S25	0	ND	0.0946	Negative
S26	0	ND	0.1079	Negative
S27	0	ND	0.1319	Negative
S28	0	ND	0.1386	Negative
S29	0	ND	0.2056	Negative
S30	0	ND	0.2071	Negative
S31	0	ND	0.2021	Negative
S32	0	ND	0.1972	Negative

Note:

DI grades were assigned as healthy = 0, mild = 1, moderate = 2, and severe = 3. ND indicates that no positive ddPCR signal was detected under the assay conditions. For regression analysis, ND values in healthy samples were treated as 0 copies/µL.

Figure S1. Schematic illustration of the MCS-free direct-fusion construct used for recombinant Trx-His-CP antigen production.

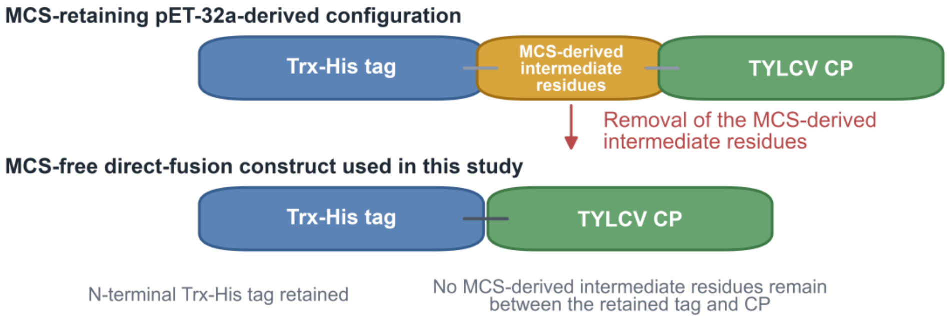

Note:

Schematic illustration of the MCS-free direct-fusion construct used for recombinant Trx-His-CP antigen production. In the MCS-retaining pET-32a-derived configuration, vector-derived intermediate residues are located between the retained N-terminal Trx-His tag and the TYLCV CP sequence. In the construct used in this study, the MCS-derived intermediate residues were removed, while the Trx-His tag was retained. This schematic is intended to clarify the cloning strategy only and does not represent direct comparative evidence of improved antigen performance.

Figure S2. Immunization protocol and preparation process of the specific pAb.

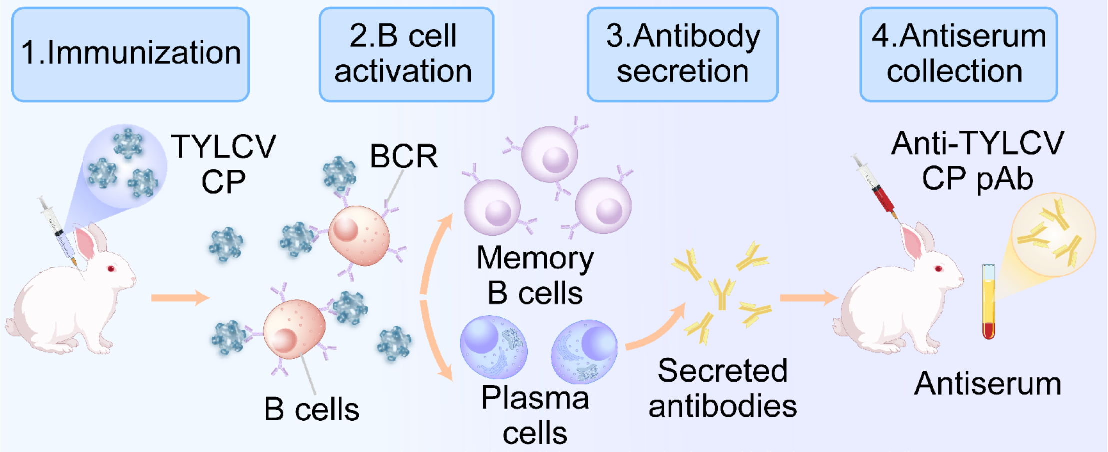

Note:

Schematic diagram of the preparation process of the TYLCV CP-specific pAb. (1) Antigen immunization: Rabbits are immunized with the purified recombinant TYLCV CP antigen via subcutaneous injection; (2) B cell activation: The antigen is recognized by B cell receptors (BCRs) in vivo, thereby activating target B cells; (3) Antibody secretion: The activated B cells differentiate into plasma cells to secrete large quantities of specific antibodies; (4) Serum collection: Rabbit antiserum is harvested to obtain the pAb against the CP.

Figure S3. Optimization of induction conditions and solubility analysis of the recombinant CP fusion protein.

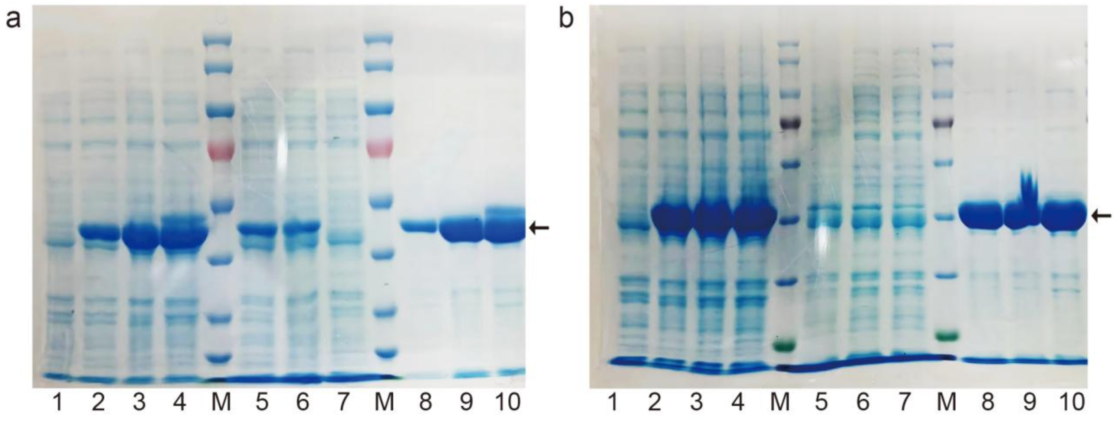

Note:

(a) Temperature gradient analysis (16°C, 20°C, and 25°C for 16 h). Lane 1: whole cells before induction; Lanes 2-4: whole cells after induction; Lanes 6-8: soluble supernatant fractions; Lanes 10-12: insoluble inclusion body pellets.

(b) Induction duration analysis (12 h, 16 h, and 20 h at 20°C). Lanes are identical to panel (a).

The black arrow denotes the target recombinant protein (48 kDa). M: protein molecular weight marker. The neighboring bands for the target protein correspond to 55 kDa and 40 kDa in panel (a) (marker profile shown in Figure S5a), and to 63 kDa and 45 kDa in panel (b) (marker profile shown in Figure S5d).

Figure S4. Densitometric purity analysis of the refolded recombinant protein using ImageJ software.

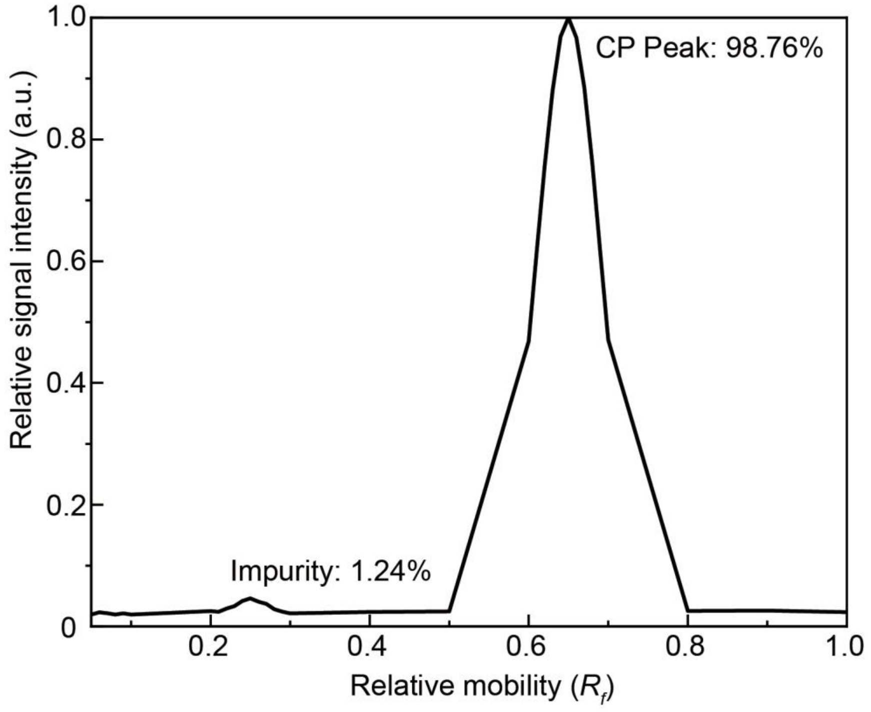

Note:
The relative mobility (R_f) profile demonstrates a target peak purity approximately 98.8%, with residual host impurities accounting for 1.24%.

Figure S5. Detailed standard band profiles for the protein molecular weight markers.

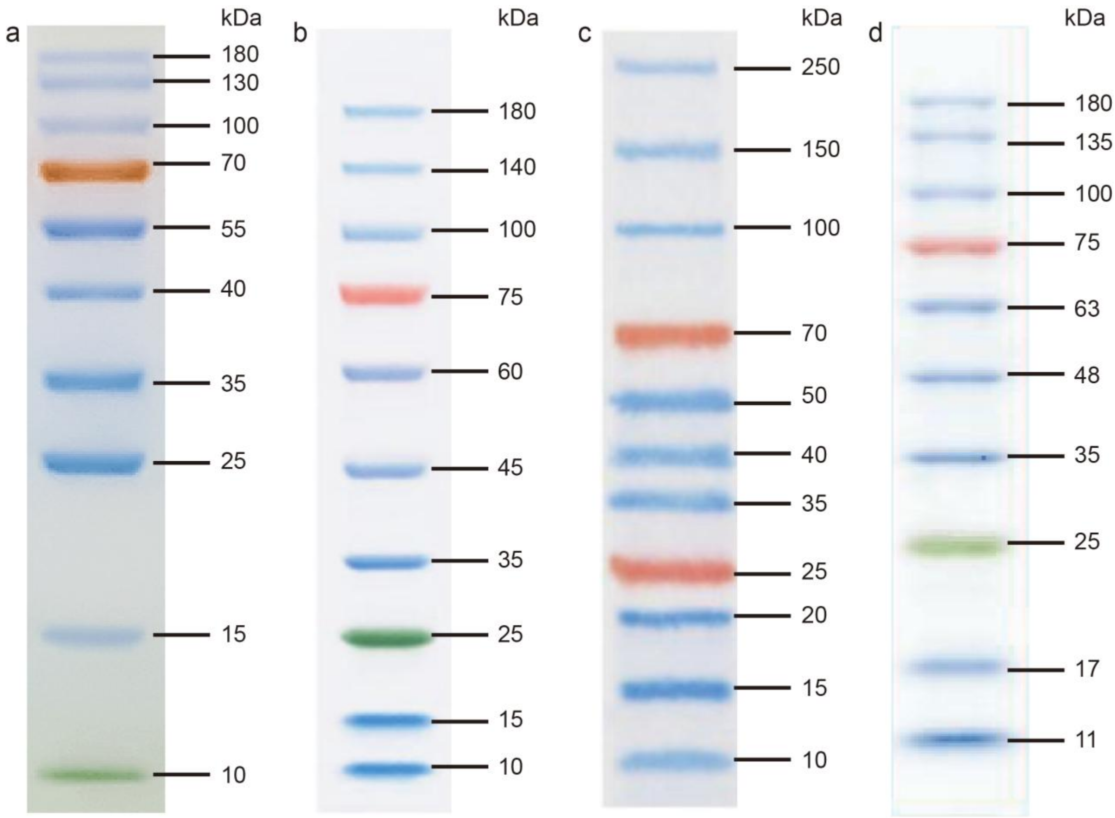

Note:

(a) Marker utilized for Figure 1a and Figure S3a.

(b) Marker utilized for Figure 1b and Figure 1c.

(c) Marker utilized for Figure 2b.

(d) Marker utilized for Figure S3b.

Figure S6. LC-MS/MS sequence coverage map of the recombinant antigen.

10 20 30 40 50

M**SDKIIHLTDDSFDTDVLK**ADGAILVDFWAEWCGPCK**MIAPILDEIADEY**

60 70 80 90 100

QGKLTVAKLNIDQNPGTAPKYGIRGIPTLLLFKNGEVAATKVGALSKGQL

110 120 130 140 150

KEFLDANLAGSGSGHMHHHHHHSSGLVPRGSGMKETAAAKFERQHMDSPD

160 170 180 190 200

LGTDDDDKAMADIGSEFMASK**RPGDIIISTPVSKVRRRLNFDSPYSSRAA**

210 220 230 240 250

VPIVQGTNKRRSWTYRPMYRKPRIYRMYR**SPDVPRGCEGPCKVQSYEQRD**

260 270 280 290 300

DIKHTGIVRCVSDVTRGSGITHRVGK**RFCVKSIYFLGKVWMDENIKKQNH**

310 320 330 340 350

TNQVMFFLVRDRRPYGNSPMDFGQVFNMFDNEPSTATVKNDLRDRFQVMR

360 370 380 390 400

KFHATVIGGPSGMKEQALVKRFFRINSHVTYNHQEAAKYENHTENALLLY

410 420 430

MACTHASNPVYATMKIRIYFYDSISNLEHHHHHH

Note:

LC-MS/MS sequence coverage map of the recombinant TYLCV CP. Peptide fragments identified by mass spectrometry are highlighted in red, representing a total sequence coverage of 85.3%. Grey letters indicate undetected regions.

Figure S7. MS/MS fragmentation spectrum of the representative high-abundance peptide INSHVTYNHQEAAK.

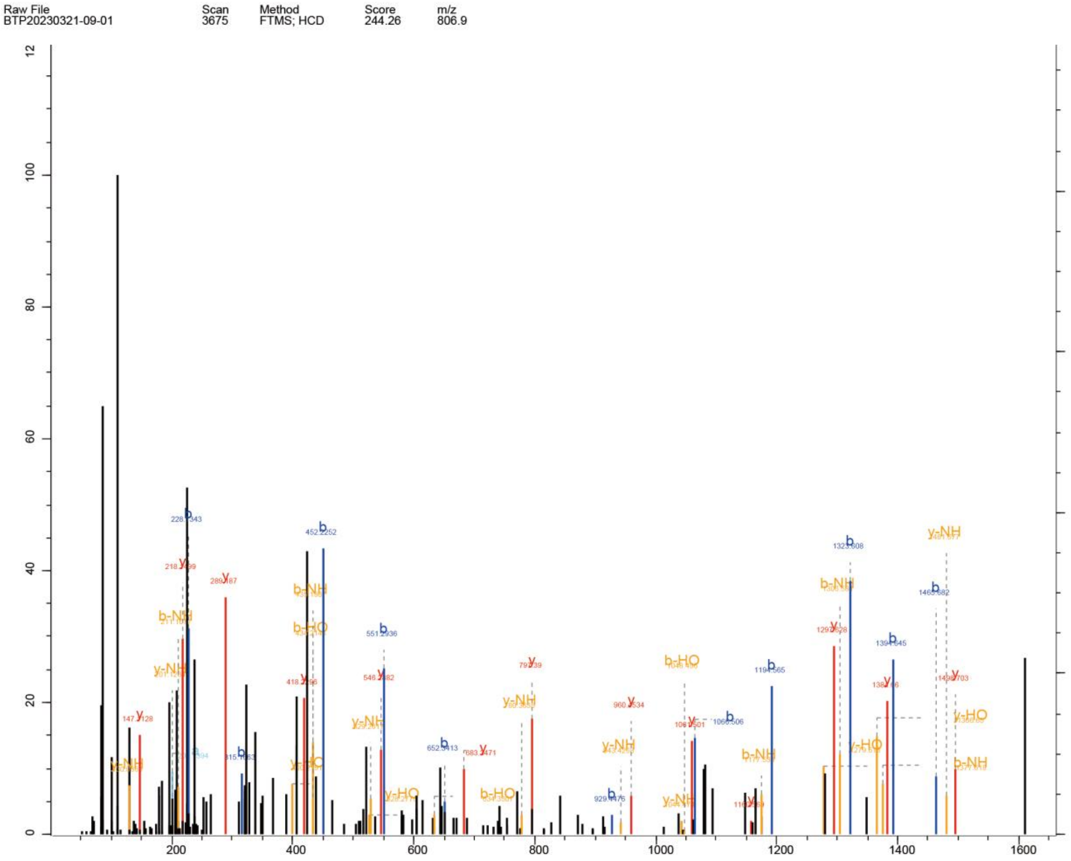

Note:

This representative peptide (MaxQuant score: 244.26, scan: 3675) corresponds to residues 208-221 of the TYLCV CP sequence. Red and blue peaks denote the observed y-ion and b-ion series, confirming the identity of the sequence.

Figure S8. Specificity control test and pAb response curves against the recombinant Trx control protein.

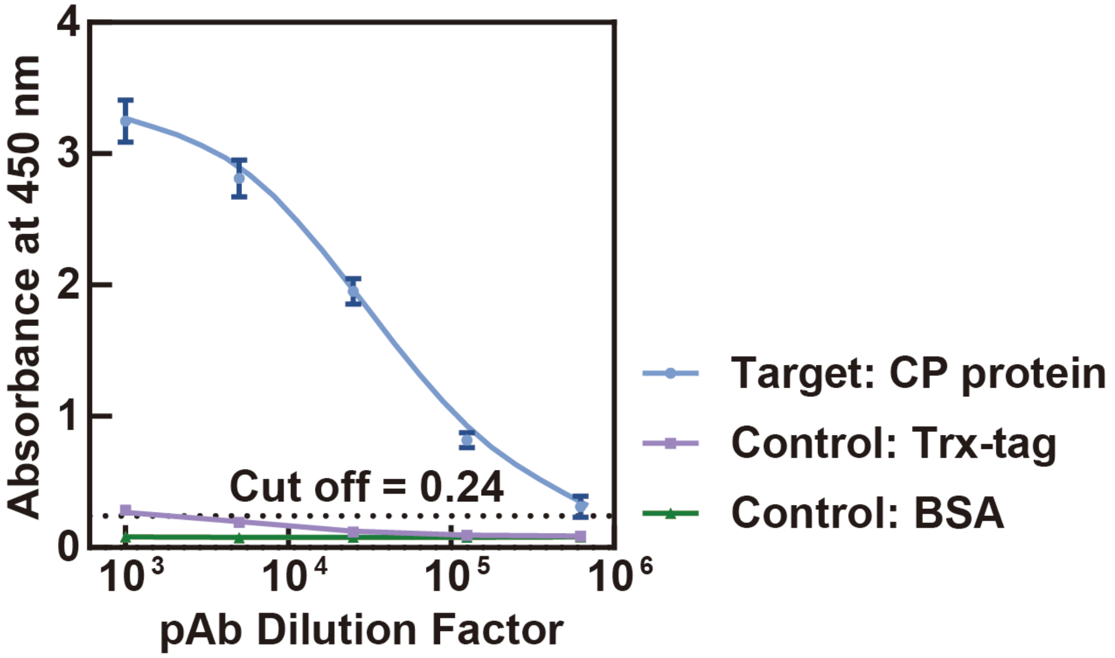

Note:

Absorbance values (OD_{450}) of the purified pAb were measured across serial pAb dilutions using the recombinant Trx-His-CP fusion antigen, recombinant Trx control protein, and BSA as coated antigens. The horizontal dashed line denotes the positive cut-off threshold of 0.24.

Figure S9. Per-residue model confidence scores (pLDDT) plot for the predicted TYLCV CP structure.

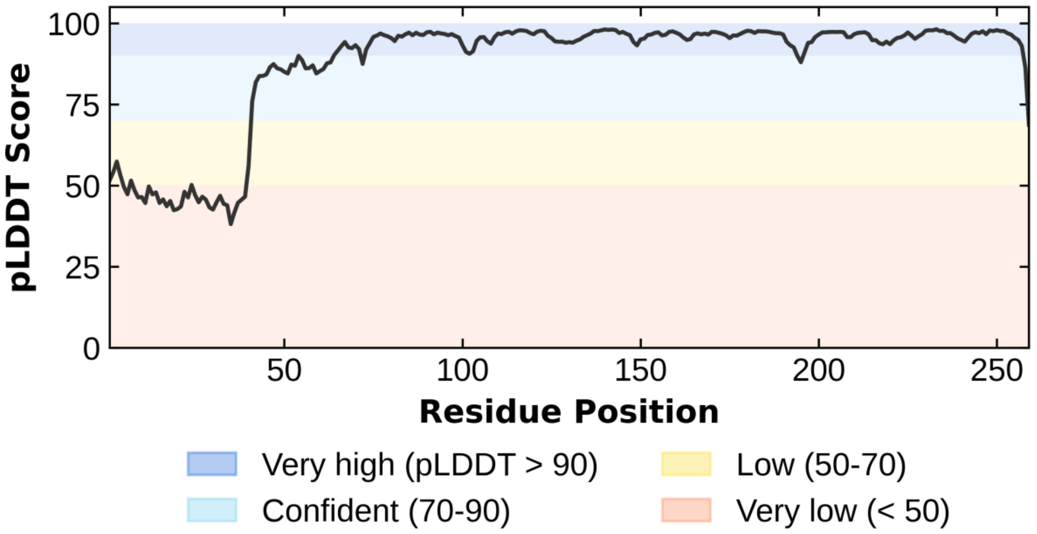

Note:

The black line indicates the pLDDT score across the full-length amino acid sequence (1-259 residues). Horizontal shaded bands represent distinct confidence levels defined by AlphaFold2: very high (pLDDT>90), confident (pLDDT=70-90), low (pLDDT=50-70), and very low (pLDDT<50). The core domain demonstrates reliable structural prediction with scores consistently above 90.
